# The oncogenic role of NF1 in gallbladder cancer through regulation of YAP1 stability by direct interaction with YAP1

**DOI:** 10.1186/s12967-023-04157-9

**Published:** 2023-05-05

**Authors:** Lingxiao Zhang, Lin Jiang, Ling Zeng, Zhaohui Jin, Xuanjia Dong, Yuhan Zhang, Litian Chen, Yijun Shu, Yingbin Liu, Ying Huang

**Affiliations:** 1 Department of General Surgery, Shanghai Key Laboratory of Biliary Tract Disease Research, Xinhua Hospital, Shanghai Jiao Tong University, Shanghai, 200092 China; 2 Department of Biliary-Pancreatic Surgery, State Key Laboratory of Oncogenes and Related Genes, Renji Hospital Affiliated to Shanghai Jiao Tong University School of Medicine, Shanghai, 200127 China; 3 School of Health Science and Engineering, University of Shanghai for Science and Technology, Shanghai, 200093 China

**Keywords:** Neurofibromin 1 (NF1), Gallbladder cancer (GBC), YAP1, Hippo pathway

## Abstract

**Background:**

Gallbladder cancer (GBC) is the most prevalent and invasive biliary tract malignancy. As a GTPase-activating protein, Neurofibromin 1 (NF1) is a tumor suppressor that negatively regulates the RAS signaling pathway, and its abnormality leads to neurofibromatosis type 1 (NF-1) disease. However, the role of NF1 playing in GBC and the underlying molecular mechanism has not been defined yet.

**Methods:**

A combination of NOZ and EH-GB1 cell lines as well as nude mice, were utilized in this study. mRNA expression and protein levels of NF1 and YAP1 were evaluated by quantitative real-time PCR (qRT-PCR), western blot (WB), and immunohistochemistry (IHC). In vitro and in vivo assays were performed to explore the biological effects of NF1 in NOZ and EH-GB1 cells via siRNA or lv-shRNA mediated knockdown. Direct interaction between NF1 and YAP1 was detected by confocal microscopy and co-immunoprecipitation (Co-IP), and further confirmed by GST pull-down assay and isothermal titration calorimetry assay (ITC). The stability of proteins was measured by western blot (WB) in the presence of cycloheximide.

**Results:**

This study showed that a higher level of NF1 and YAP1 was found in GBC samples than in normal tissues and associated with worse prognoses. The NF1 knockdown impaired the proliferation and migration of NOZ in vivo and in vitro by downregulating YAP1 expression. Moreover, NF1 co-localized with YAP1 in NOZ and EH-GB1 cells, and the WW domains of YAP1 specifically recognized the PPQY motif of NF1. The structural modeling also indicated the hydrophobic interactions between YAP1 and NF1. On the other hand, YAP1 knockdown also impaired the proliferation of NOZ in vitro, phenocopying the effects of NF1 knockdown. Overexpression of YAP1 can partially rescue the impaired proliferation in NF1 stably knockdown cells. In mechanism, NF1 interacted with YAP1 and increased the stability of YAP1 by preventing ubiquitination.

**Conclusions:**

Our findings discovered a novel oncogenic function of NF1 by directly interacting with YAP1 protein and stabilizing YAP1 to protect it from proteasome degradation in NOZ cells. NF1 may serve as a potential therapeutic target in GBC.

**Supplementary Information:**

The online version contains supplementary material available at 10.1186/s12967-023-04157-9.

## Background

Gallbladder cancer (GBC) is a regional-prevalent and aggressive carcinoma with rare morbidity and high mortality. It is the most common and invasive tumor of the biliary tract malignancies [1]. The incidence rate of GBC is higher in Asia compared to Western countries, but unfortunately, most cases are diagnosed at advanced stages, making surgical resection impossible. Only a small proportion of patients with early-stage GBC are eligible for surgery, and most adjuvant therapies have a low response rate [2–4]. Due to the characteristic pattern of late diagnosis, ineffective treatment, and poor overall prognosis associated with GBC [5, 6], the mean survival ranges from 13.2 to 19 months [7, 8]. In this regard, it is crucial to identify novel genes that could improve GBC management.

Neurofibromin 1 (NF1) is a GTPase that converts GTP to GDP. It functions as a tumor suppressor by negatively regulating Ras proteins, converting Ras from an active GTP-bound state to an inactive GDP-bound state [9]. NF1 can, therefore, act as a shutdown signal for all vertebrate RAS proteins, including KRAS, NRAS, HRAS, MRAS, RRAS, and RRAS2 [10]. It is primarily expressed in the nervous system [11]. Germline mutations in this gene can cause neurofibromatosis type 1 (NF-1), an autosomal dominant monogenetic disease affecting approximately one in every 3,000 individuals worldwide [12, 13]. Loss of function mutations in NF1 lead to sustained activation of intracellular RAS-GTP, prolonged activation of the RAS/RAF/MAPK signaling pathway, uncontrolled cell proliferation, malignancy, and tumor growth [14, 15]. NF1 is a large protein with 2819 amino acid residues. Recent studies have analyzed the structure of NF1, demonstrating that in addition to the GTPase domain, it contains a PH domain that interacts with SPRED protein to attach to the inner surface of the cell membrane [16–18]. The remaining N-terminal and C-terminal domains serve as scaffolds and stabilize the protein. While research on NF1 has primarily focused on its GTPase domain (also known as the GTPase-activating protein-related domain, GRD), the HEAT domains at the N- and C-terminals have been less studied.

Interestingly, somatic mutations in NF1 are observed in 5–10% of cancers, including sarcomas such as malignant peripheral nerve sheath tumors (MPNST), brain and breast cancers, juvenile myelomonocytic leukemia, and Watson syndrome [19–21]. In addition, mutations in the NF1 gene have been identified in many sporadic malignant tumors not associated with NF-1 [22]. It has also been reported that individuals with NF1 may have an increased susceptibility to other neoplasms [13]. Recent studies have suggested that NF1 may also play a role in breast tumors [23], colorectal cancer [24], pancreatic ductal adenocarcinoma [25], gastrointestinal stromal tumor (GIST), and neoplasm of the bile duct [26]. Despite these findings, it remains unclear how NF1 contributes to the pathogenesis of these malignancies, and its role in GBC remains poorly understood.

In this study, we investigated the role of NF1 in GBC both in vitro and in vivo. Our results demonstrated that NF1 was significantly upregulated in GBC and functioned as an oncogene, promoting tumor growth and migration. Notably, knockdown of NF1 led to a marked inhibition of tumor growth. Further investigation revealed that the C-terminal PPQY motif of the NF1 protein interacted directly with the tandem WW domains of YAP1 (Yes-associated protein 1), thus preventing YAP1 from proteasomal degradation. YAP1 is a transcriptional coactivator of the Hippo signaling pathway and has been implicated in the development and progression of various types of cancer, making it a potential therapeutic target [27, 28]. Knockdown of YAP1 in NOZ cells mimicked the effects observed upon NF1 knockdown. Furthermore, the NF1 fragment containing the PPQY motif was able to partially rescue the phenotype induced by NF1 knockdown. Our findings provide new insights into the possibility of targeting YAP1 through NF1 as a potential therapeutic strategy for GBC.

## Material and methods

### Clinical specimens and immunohistochemistry (IHC)

Human GBC samples and adjacent benign gallbladder tissues were obtained from patients who underwent cholecystectomy without receiving preoperative chemotherapy, radiotherapy, or androgen therapy at the Department of General Surgery, Xinhua Hospital, School of Medicine, Shanghai Jiao Tong University, between 2016 and 2021. Written informed consent was obtained from all participants. This study was approved by the ethics committee of Xinhua Hospital. Immunohistochemical staining (IHC) was performed on each tissue sample, which was fixed in 4% formalin immediately after removal and embedded in paraffin for IHC analysis using the standard staining procedure [29]. The sections were observed via a microscopic device (Leica), and the expression levels of ki67 and YAP1 were evaluated by the Image J program. The scoring criteria of NF1 or YAP1 expression level in IHC were listed in Additional file 1: Table S1.

### Cell culture and chemicals

The NOZ cell line was obtained from the Health Science Research Bank (Osaka, Japan). The following cells were acquired from the Chinese Academy of Sciences Cell Bank (Shanghai, China): HEK 293 T, GBC-SD, EH-GB1, SGC996, and HELA. Incubation was carried out at 37 °C with 5% CO_2_ in a medium containing 10% fetal bovine serum (Gibco) and 1% penicillin G/ streptomycin. The NOZ cells were cultured using Williams (Gibco), while HEK 293 T, GBC-SD, EH-GB1, and HELA cells were cultured using DMEM (Gibco), and SGC-996 cells were cultured with RPMI 1640 (Gibco). Proteasome inhibitors MG132 (HY-13259) and cycloheximide (CHX, HY-12320) were provided by MedChemExpress.

### RNA extraction and quantitative real-time PCR (qRT-PCR)

Cell lines were employed to extract RNA using the TRIzol reagent (Invitrogen, USA). PrimeScript RT Reagent Kit (Takara) was employed to reverse RNA transcription to cDNA. qRT-PCR was performed on a Real-Time PCR instrument (Applied Biosystems) with SYBR Premix Ex TaqII (Takara). The relative quantities of RNA were calculated by comparing Ct values and were standardized to GAPDH. The primer sequences utilized in this study were listed in Additional file 2: Table S2.

### Western blot

The western blot analysis involved the use of the following antibodies at specific dilutions: anti-NF1 (1:1000, Proteintech), anti-YAP1 (1:4000, Proteintech), anti-Ubiquitin (1:20000, Proteintech), and anti-β-actin (1:50000, ABclonal). Per the manufacturer's instructions, the NF1 samples were analyzed using an automated capillary-based size sorting system (Wes, ProteinSimple) [30, 31]. Data analysis was carried out using the built-in Compass software (ProteinSimple).

### Cell transfection

NF1 was silenced with small interfering RNAs (siRNAs) with Lipofectamine 3000 (Thermo Fisher), following the manufacturer’s protocol. The target sequences were si-NF1-1 (target sequence, GGCCUAGCAAUCGCUUUAATT); si-NF1-2 (target sequence, GGCAGAUAAAGCAGAUAAUTT). si-YAP1-1 (target sequence, CCACCAAGCTAGATAAAGA); si-YAP1-1 (target sequence, GAGATGGAATGAACATAGA). NF1 and YAP1 siRNAs were obtained from RiboBio (Guangzhou, China). The lv-shRNAs targeting NF1 were synthesized according to the sequences of si-NF1-1 and si-NF2-1 and inserted into the GV493 vector. The full-length YAP1 and NF1 spanning residues 2560–2818 were cloned into the pCMV plasmid vector. Empty vectors were used as control. NOZ cells were infected by concentrated lentivirus at a multiplicity of infection (MOI) of 10 for 48 h, using HiTransG A as recommended by the manufacturer’s protocol. Stable cell lines were generated by puromycin (1 μg/ml) treatment for one week. Transfection efficiency was verified by qRT-PCR and western blotting.

### CCK-8 assay and colony formation

Cell proliferation was assessed using CCK-8 assays. Approximately 2000 cells per well were seeded into 96-well plates. Next, 10 μL of the CCK-8 reagent (YEASEN) was added to each well, and the microplate was incubated for 2 h at 37 °C. The OD_450_ was measured using a microplate reader (Multiskan Sky, Thermofisher) every 24 h for 96 h.

Cells were seeded into 6-well plates at 2 × 10^3^ cells/well density for the colony formation assay and cultured in media containing 10% fetal bovine serum (FBS). After two weeks, the cells were treated with 4% polyformaldehyde (PFA) and stained with 0.1% crystal violet. The number of visible colonies was counted.

### Wound healing and transwell migration assay

To perform the wound healing assay, cells were seeded into 6-well plates and allowed to grow to a more than 90% confluency. The monolayer was then uniformly wounded using a 200-μL pipette tip. After washing with fresh medium, the cells were incubated in a serum-free medium for 24 h, and images were taken from 5 random fields of view using a microscope. The wound closure percentage was evaluated by comparing the changes in the wound area before and after 24 h.

For the Transwell migration assay, 24-well plates with 8 μm chamber inserts (Corning Life Science) were used. A total of 1 × 10^5^ cells were seeded in the upper chamber with a serum-free medium in triplicate. Medium containing 10% FBS was added to the lower chamber as a chemo-attractant. After incubation for 24 h, the cells in the chamber were removed by cotton swabs, and the cells below the membrane were fixed with 4% PFA, stained with 0.1% crystal violet for 10 min, and counted.

### In vivo* mouse models*

Male BALB/c nude mice, aged 4 weeks and weighing 18–22 g, were procured from the Shanghai Laboratory Animal Center of the Chinese Academy of Sciences in Shanghai, China, and maintained under specific pathogen-free conditions on autoclaved mouse chow. All animal procedures were authorized by the Ethics Committee of the Xinhua Hospital Affiliated to Shanghai Jiao Tong University, School of Medicine (No. XHEC-F-2022-080). For the tumorigenesis studies, five mice were assigned to each group and injected subcutaneously with 1 × 10^6^ NOZ cells, which had been stably transfected with the expression vectors of interest and suspended in 100 μl PBS, into the right axilla. The tumor size was estimated weekly using the formula: Volume = π/6 × width^2^ × length. After four weeks, the mice were euthanized, and the tumors were excised, weighed, and sent for further analysis.

### Immunofluorescence staining and laser confocal microscopy

The cells were fixed with 4% PFA and permeated in 0.1% Triton-X-100. The cells were then blocked using 3% BSA, followed by overnight incubation at 4 °C with the primary antibodies. Labeled secondary antibodies were added and incubated at room temperature, away from light. DAPI was utilized for cell counterstaining, and the immunofluorescence in situ was visualized with a laser confocal microscope (Leica TCS SP8 II). The colocalization analysis was performed with the help of the image processing software Image J as described previously [32].

Primary antibodies were used as follows: NF1 (1:100, Proteintech) and YAP1 (1:500, Proteintech). Alexa Fluro 647-conjugated Goat anti-rabbit IgG (1:500, BBI-biotech) and Alexa Fluro 488-conjugated Goat anti-mouse IgG (1:500, BBI-biotech) were employed as secondary antibodies.

### Co-immunoprecipitation

HEK 293 T cells were seeded in six-well plates at approximately 1 × 10^6^ cells/ml density and transfected with the corresponding recombinant plasmids using HighGene transfection reagent (ABclonal). After 48 h of transfection, cells were harvested and lysed. The resulting cell lysates were incubated overnight at 4 °C with anti-FLAG affinity beads (Smart-Lifescience) supplemented with 1 mg/ml BSA. The beads were washed three times with washing buffer (50 mM Tris pH 8.0, 150 mM NaCl, 0.1% Triton, and 5 mM EDTA), boiled in 50 μl 2 × SDS loading buffer, and then subjected to western blot analysis.

### GST pull-down assay

For GST pull-down assays, GST, GST-NF1^PPQY^ (residues 2665–2687), GST-NF1^AAAA^ (GST-NF1^PPQY^ with the four PPQY residues replaced by alanine), His-Sumo-YAP1^WW1^ (residues 174–204), and His-Sumo-YAP1^WW2^ (residues 227–266) were purified. Increasing amounts of His-Sumo-YAP1^WW1^ and His-Sumo-YAP1^WW2^ were added to GST-NF1^PPQY^ and GST-NF1^AAAA^, prebound on Glutathione Sepharose beads (Cytiva), and incubated in a binding buffer (20 mM Tris, pH 8.0, 150 mM NaCl) for 2 h with rotation at 4 °C. Purified GST protein was used as a control. After washing the beads three times with the binding buffer, the bound proteins were eluted with 10 mM GSH. The eluates were subjected to 15% SDS–PAGE and stained with Coomassie blue.

### Isothermal titration calorimetry assay

The NF1^PPQY^ peptide (residues 2671–2680: EESPPQYQTS) used in ITC assays were synthesized (Scilight-Peptide, China). All measurements were performed at 25 °C using a PEAQ-ITC (Microcal). The experiments were carried out by injecting 2 μl of 10 mM NF1^PPQY^ into the sample cell containing 1 mΜ YAP1^WW1^/YAP1^WW2^ solution, 10 mM Tris, pH 8.0, and 100 mM NaCl for 20 injections. The binding isotherms and standard deviation were fitted to a one-site binding model using the Origin 8.0 software (Microcal).

### Structure modeling

Structural models of the interactions between YAP1 tandem WW domains and the NF1 PPQY motif were generated based on the crystal structure of YAP1 WW domains in complex with Dendrin (PDB 6JK1). The models were further validated using AlphaFold. Structure figures were presented using the Pymol program (The PyMOL Molecular Graphics System, Version 2.0 Schrödinger, LLC).

### Cycloheximide chase assay

To analyze YAP1 protein levels, NOZ cells were treated with 50 μg/mL cycloheximide (CHX) for 4 h before lysates were collected at different time points and analyzed by western blot. The integrated density of immunoblotting was measured using Image J to obtain the YAP1 run chart.

### Statistical analysis

All statistical analyses were performed using SPSS 25.0 software. Each experiment was repeated three times, and the data are presented as the mean ± SD unless otherwise stated. The survival analysis was performed using COX regression. An unpaired t-test was utilized to compare the statistical difference. Pearson chi-square test was employed to analyze the association between NF1 and YAP1 expression. *P* value less than 0.05 was considered statistically significant.

## Results

### NF1 was frequently mutated and associated with poor prognosis in GBC

**Fig. 1:**
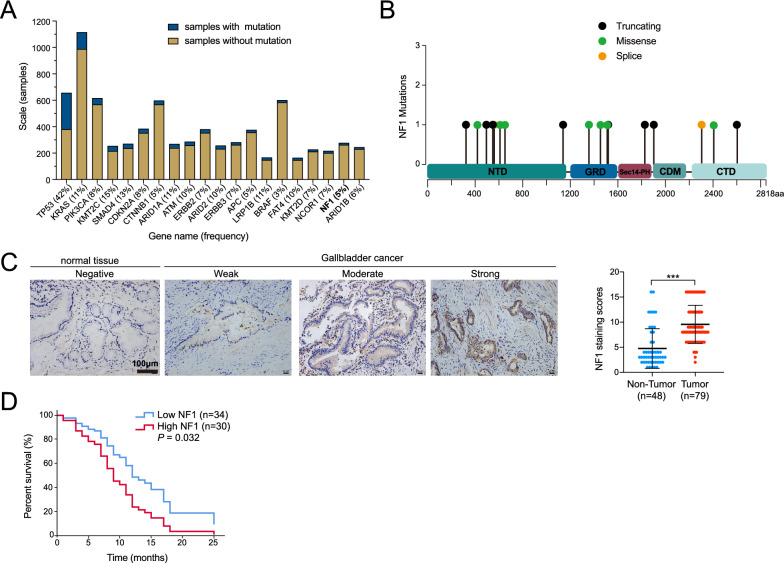
NF1 was among the top mutated genes and correlated with poor prognosis in gallbladder cancer. **A** NF1 was identified as one of the top 20 muted genes in gallbladder carcinoma from the COSMIC database. **B** The somatic mutations of NF1 in GBC were presented from the cBioPortal database, and the domain organization of NF1 was shown: NTD (N-terminal domain, dark teal), GRD (GAP-related domain, dark blue), Sec14-PH (secretory protein 14-pleckstrin homology-like module, raspberry), CDM (central dimerization module, teal), CTD (C-terminal domain, light blue). **C** Left panel: representative images of NF1 expression in GBC tissues and adjacent non-tumor tissues using IHC staining. Right panel: The IHC staining scores of NF1 were higher in GBC tissues than in non-tumor tissues. ***P < 0.001, n = 79.** D** NF1 differently expressed were subjected to further multivariate Cox regression analysis to evaluate correlation with overall survival of GBC patients (*P* = 0.032, n = 64)

To investigate the potential role of NF1 in GBC, a comprehensive analysis of NF1 expression in the tumor spectrum was conducted through the TCGA and GTEx databases. The expression level of NF1 in digestive system tumors, including CHOL (Cholangiocarcinoma), LIHC (Liver hepatocellular carcinoma), PAAD (Pancreatic adenocarcinoma), and STAD (Stomach adenocarcinoma), was found to be higher than that in the adjacent non-cancerous tissue (Additional file 3: Fig. S1A). Since the somatic mutations of NF1 have been detected in various cancers, somatic mutations of NF1 in GBC were investigated. According to the Catalogue of Somatic Mutation in Cancer (COSMIC) database, NF1 is among the 20 most frequently mutated genes in GBC, with a somatic mutation frequency of 5%, based on the sequencing of nearly 300 samples (Fig. 1A). Furthermore, data from cBioPortal for Cancer Genomics indicate that NF1 mutations, including truncation, splicing, and missense mutations, are distributed throughout the NF1 protein (Fig. 1B). Moreover, mutations in NF1 were also observed in other digestive system tumors. In CHOL and STAD, many mutations were found at the C-terminal part of the protein, not restricted to the GRD catalytic domain (Additional file 3: Fig. S1B).

To validate the presence of NF1 protein in GBC, immunohistochemical staining (IHC) was employed. NF1 protein expression was found to be significantly higher in GBC tissues than in benign tissues (*P* < 0.001; Fig. 1C). To further investigate the relationship between NF1 expression levels and GBC progression, 64 GBC patients were categorized into low and high expression groups based on the median expression level of NF1 as a cutoff point. The survival analysis was performed using COX regression, considering variables such as age, gender, and tumor-node-metastasis (TNM) stage, revealing that the high NF1 expression group had significantly lower survival compared to the low expression group with a hazard ratio of 1.99 (95% confidence interval, 1.06 to 3.75, *P* = 0.032) (Fig. 1D). These results indicated a potential role of NF1 in the development of GBC.

### *NF1 promoted the proliferation and migration of NOZ cells *in vitro

**Fig. 2:**
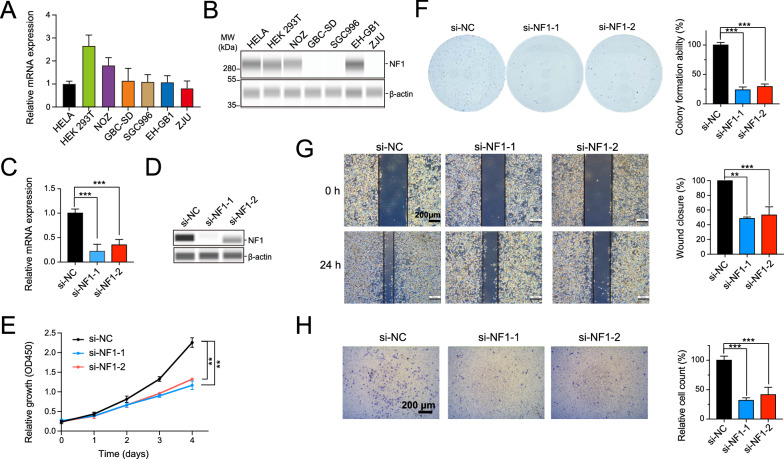
Knockdown of NF1 led to impairment of proliferation and migration of GBC in *vitro*. **A** and **B** The mRNA and protein expression levels of NF1 in HEK 293 T, HELA, and GBC cell lines (The mRNA expression level in HELA cell was regarded as control). **C** and **D** Knockdown of NF1 in NOZ cell line was confirmed at the mRNA and protein level by qRT-PCR and western blotting. *** *P* < 0.001. **E** and **F** CCK-8 and colony formation assays were applied to determine the proliferation of NOZ after NF1 depletion. **G** and **H** Would healing assay and transwell assays were conducted to measure the migration of NOZ after NF1 depletion

Interestingly, although the levels of NF1 mRNA were found to be similar across the various GBC cell lines when HELA cells were used as a standard (Fig. 2A), the NF1 protein expression was only observed in NOZ and EH-GB1 cell lines (Fig. 2B). An investigation into NF1’s specific role in cell proliferation and tumorigenesis was carried out by knocking down NF1 in NOZ cells by RNAi, following data mining result indicating that high NF1 expression is associated with a poor prognosis of GBC. The knockdown and transfection efficiency were validated using qRT-PCR and western blot analysis (Fig. 2C, D). After four days of culture, knockdown of NF1 resulted in a significant decrease in cell proliferation as measured by the CCK-8 assays (Fig. 2E). Additionally, there was a marked reduction in colony formation (Fig. 2F). The migration ability of NOZ cells in vitro was investigated using wound healing and transwell migration assays, which showed that NF1 knockdown significantly inhibited the migration ability of NOZ cells compared with the negative control (Fig. 2G, H). Moreover, qPCR analysis was also performed to verify the knockdown efficiency of NF1 in EH-GB1 cells (Additional file 4: Fig. S2A). Cell proliferation and colony formation assays in EH-GB1 cells then yielded similar results as in NOZ cells (Additional file 4: Fig. S2B, C), further confirming the role of NF1 in GBC cell growth.

### *NF1 promoted NOZ cell proliferation *in vivo

**Fig. 3:**
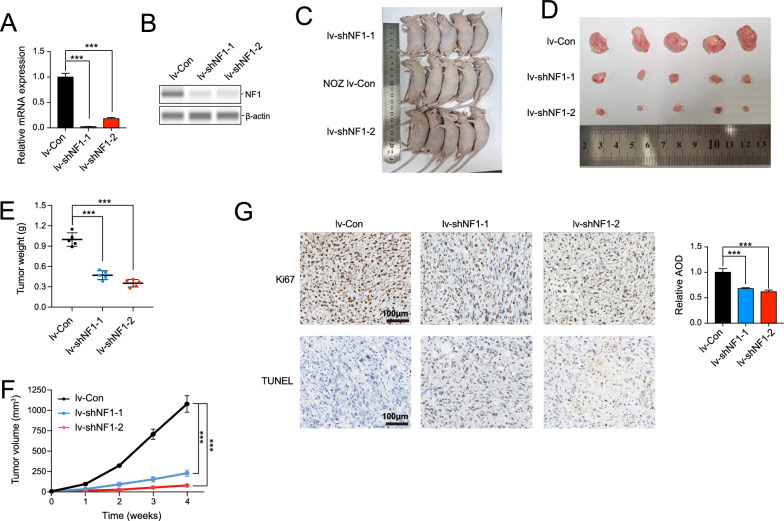
Knockdown of NF1 led to impairment of proliferation of GBC in *vivo*. **A** and **B** The mRNA and protein expression level of NF1 in NOZ cells transfected with lv-Con, lv -shNF1-1, and lv -shNF1-2. **C** The nude mice used for tumor growth in vivo were shown. **D** and **E** Tumor weight was measured after mice were sacrificed and the tumors were resected. Compared with lv -Con groups, knockdown of NF1 suppressed the tumor growth in nude mice. *** *P* < 0.001 **F** Tumor growth curves were plotted. **G** The expression of Ki67 and TUNEL by IHC staining

In order to investigate the effect of NF1 on NOZ cell growth in vivo, two lentiviral vectors expressing shRNAs (lv-shNF1-1 and lv-shNF1-2) were constructed to stably knock down NF1 in NOZ cells (Fig. 3A, B). To establish the cell line-derived xenograft (CDX) models, NOZ cells transduced with lv-shNF1-1, lv-shNF1-2, and lv-con (the vector) were subcutaneously injected into nude mice (Fig. 3C). The tumor volume and weight of the mice injected with NOZ cells containing either lv-shNF1-1 or lv-shNF1-2 were significantly reduced compared to the control mice (Fig. 3D, E), as was the tumor growth curve (Fig. 3F). Moreover, Ki67 and TUNEL staining were performed on the tissue sections from the CDX mouse models to verify the oncogenic role of NF1. Upon NF1 depletion, Ki67 staining indicated decreased proliferation compared to controls (Fig. 3G), and TUNEL staining showed increased apoptosis (Fig. 3H). These results indicated that NF1 exerted oncogenic properties in GBC by promoting the proliferation and migration of NOZ cells.

### The PPQY motif of NF1 was recognized by both WW domains of YAP1

**Fig. 4:**
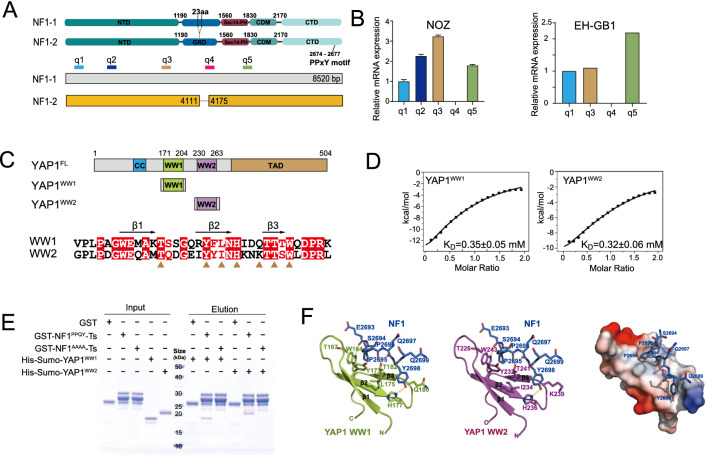
The PPQY motif in NF1 interacted directly with the WW domains of YAP1. **A** Alternatively two NF1 spliced transcript variants encoding different isoforms have been presented in this diagram, and the sites of qRT-PCR primers were marked in different colors. **B** NF1-2 was the main isoform in NOZ and EH-GB1, according to the detection of different isoforms of NF1 by qRT-PCR. **C** Domain architectures of YAP1 and the amino-acid residue numbers were indicated. The domain names were abbreviated within the respective colored regions: CC (Coiled coil, sky blue), WW1 (Green yellow), WW2 (Medium purple), and TAD (Transactivation domain, light brown). Sequence alignment of WW domains in YAP1 was presented by Clustal W. **D** Dissociation constant between PPQY and WW domains in YAP1 as analyzed by ITC assay. Quantification of the dissociation constant (K_D_) of the interaction between NF1^PPQY^ and YAP1^WW^, as measured by an isothermal titration calorimetry assay. The K_D_ value was indicated. **E** NF1^PPQY^ directly interacted with WW1 and WW2, as shown by GST Pull-down assay. SDS–PAGE showed pull-down results of the NF1 and YAP1 by either GST-tagged NF1^PPQY^ or NF1^AAAA^, respectively, compared to a GST control. 1 μg of each input protein was loaded on the left. Positions of molecular weight markers are indicated in kDa. **F** Left: Cartoon representation of NF1^PPQY^ (blue) in complex with YAP1^WW1^ (green) or YAP1^WW2^ (purple). Right: Electrostatic potential surface analysis showed the hydrophobic interactions between YAP1 WW1 and the PPQY motif

There are three main alternatively spliced isoforms of NF1, of which isoforms 1 (NF1^23aIN^) and 2 (NF1) are the most common. In isoform 2, exon 23 was excluded, resulting in a deletion of 23 residues in the GRD domain [16]. To ascertain the dominant isoform of NF1 in NOZ and EH-GB1 cells, five pairs of primers were designed at suitable DNA sites, and qRT-PCR assays were conducted (Fig. 4A). The results indicated that isoform 2 was the predominant isoform in both NOZ and EH-GB1 (Fig. 4B). Thus, the NF1 protein may possess an oncogenic function independent of its GTPase activity, which typically inactivates RAS and suppresses tumorigenesis. With 2819 amino acids, the full-length cryo-EM structure of NF1 shows that the GTPase domain accounts for only 1/10 of the protein, while the rest acts as a scaffold and is likely to engage in protein–protein interactions [17]. A recent study reported that nuclear YAP1 was elevated in Schwann cells of head and neck plexiform neurofibroma (pNF) tumor tissues, and knockdown of NF1 in Schwann cells resulted in decreased levels of phosphorylated YAP1 and increased nuclear YAP1, suggesting that NF1 may influence YAP1 localization [33].

YAP1 contains two WW domains that recognize the PPQY motif (Fig. 4C). Intriguingly, a PPQY motif is located at the C-terminus of NF1 (Fig. 4A). To determine whether a direct interaction exists between NF1 and YAP1, GST pull-down experiments were conducted. GST-NF1, which contains a PPQY motif, could recruit both WW1 and WW2 domains of YAP1, whereas GST protein as control could not (Fig. 4E). Furthermore, the substitution of the PPQY motif with four alanine residues failed to pull down WW1 and WW2 (Fig. 4E). Additionally, isothermal titration calorimetry (ITC) was performed to determine the affinity of NF1 for YAP1 WW domains. The dissociation constants of NF1 peptides to WW1 and WW2 were found to be 0.32 mM and 0.346 mM, respectively (Fig. 4D). A model of the interactions was generated based on AlphaFold as well as the previously reported crystal structure of YAP1 tandem WW domains in complex with Dendrin protein (PDB 6JK1) (Fig. 4F and Additional file 5: Fig. S3). The findings suggested that NF1 and YAP1 exhibited a direct interplay in vitro through recognition of the PPQY motif of NF1 by the WW domains of YAP1.

### *NF1 interacted with and colocalized with YAP1 *in vivo

**Fig. 5:**
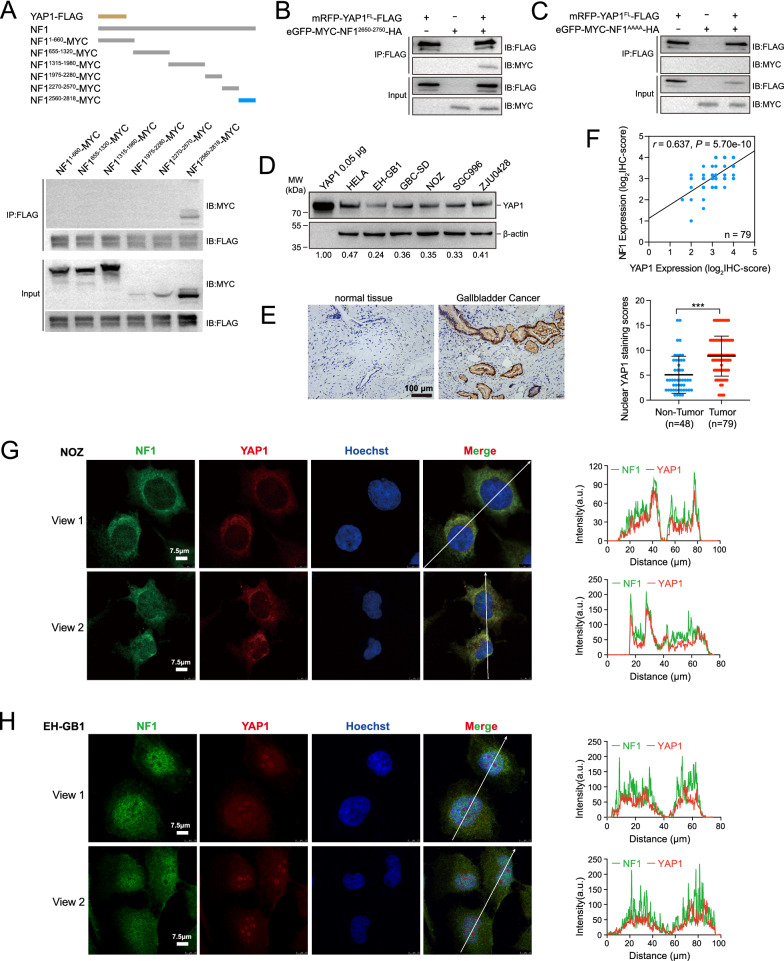
NF1 interacted with YAP1 in NOZ cells. **A** Top panel, fragments of recombinant plasmids, including YAP1-FALG and NF1-MYC. The amino-acid residue numbers were indicated for each protein. Bottom panel, co-immunoprecipitation results of YAP1 and different segments of NF1. The interacting regions between NF1 and YAP1 were mapped using Co-IP assays. **B** Co-immunoprecipitation of YAP1 and NF1^2560−2818^.** C** Co-immunoprecipitation of YAP1 and NF1^AAAA^. NF1^AAAA^ represented the alanine substitution of the PPQY motif in NF1^2560−2818^. **D** The protein expression levels of YAP1 in HELA and GBC cell lines. Bottom numbers: the protein levels of YAP1 relative to β-actin were quantified.** E** The IHC staining scores of nuclear YAP1 were higher in GBC tissues than in adjacent non-tumor tissues. ***P < 0.001.** F** Correlation analysis between NF1 and YAP1 expression in IHC samples. **G** and **H** Left panel, representative immunofluorescence images of YAP1 (red) and NF1 (green) in situ confirmed colocalization in NOZ cells (**G**) and EH-GB1 cells (**H**). Scale bar, 7.5 μm. Right panel, plots of the red and green pixel intensities along the white arrow in the merge panel

To further investigate the interaction between NF1 and YAP1 in vivo, six fragments covering full-length NF1 were constructed, each tagged with a Myc tag (Fig. 5A). Co-IP assays were performed on NOZ cells transfected with Myc-NF1 fragments and FLAG-YAP1. Only the NF1 fragment containing the PPQY motif exhibited an interaction with FLAG-YAP1 (Fig. 5B). The substitution of four alanine residues for the PPQY motif resulted in the abolishment of these interactions, indicating that YAP1 recognizes NF1 via the PPQY motif (Fig. 5C).

The interaction between endogenous YAP1 and endogenous NF1 was also examined. YAP1 expression was measured in five GBC cell lines, and 0.05 μg of purified YAP1 protein was added as a control for western blot analysis. The expression of YAP1 was found to be within a two-fold range in all GBC cell lines, unlike NF1 (Fig. 5D). The presence of YAP1 protein was detected using IHC, and the results were consistent with NF1, showing significantly higher nuclear expression of YAP1 in GBC tissues than in benign tissues (*P* < 0.01; Fig. 5E). A significant correlation between NF1 and YAP1 expression in GBC samples was found Upon analysis of the IHC data, with an R value of 0.637 and a p value of 5.70e-10, respectively (Fig. 5F). Furthermore, analysis of gastrointestinal tumor data from the TCGA and GTEx database also revealed a statistically significant correlation between the expression of NF1 and YAP1 in CHOL (n = 36, R = 0.687, p = 3.70e-6), LIHC (n = 371, R = 0.690, p = 7.98e-54), PAAD (n = 179, R = 0.538, p = 1.87e-32), and STAD (n = 415, R = 0.640, p = 5.09e-22) (Additional file 6: Fig. S4). While correlation does not necessarily imply causation, these findings suggested that the relationship between NF1 and YAP1 in NOZ cells may also occur in primary GBC tumors and could potentially be a common feature in gastrointestinal tumors.

The localization of endogenously expressed NF1 and YAP1 was observed in NOZ and EH-GB1 cells using laser scanning confocal microscopy. YAP1 and NF1 were colocalized in both cells as calculated by Colocalization Finder in Image J. In NOZ cells, both NF1 and YAP1 were mainly localized in the cytoplasm, while in EH-GB1 cells, YAP1 was also observed to be localized partially in the nucleoli (Fig. 5G, H and Additional file 7: Fig. S5).

### *NF1 stabilized YAP1 and hindered its ubiquitination *in vitro

**Fig. 6:**
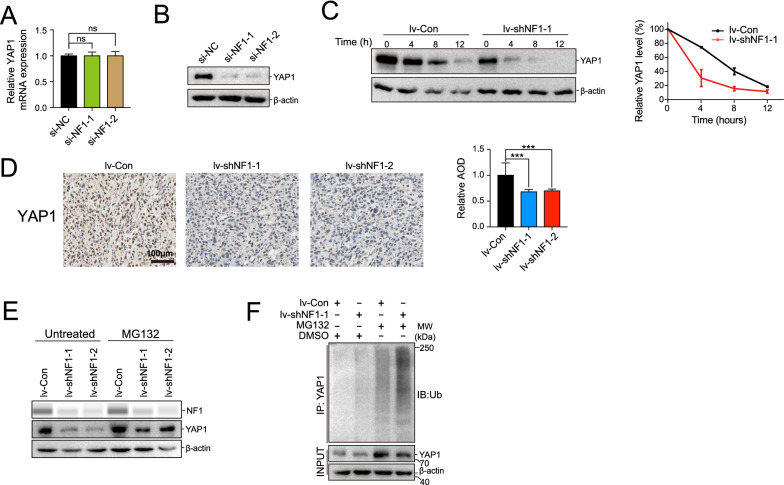
NF1 stabilized YAP1 and hindered its ubiquitination. **A** The mRNA level of YAP1 was stably expressed in the NOZ cell line with knockdown of NF1. **B** The protein level of YAP1 was reduced in the NOZ cell line with knockdown of NF1.** C** CHX chase assay was performed in the lv-Con and lv-shNF1 groups. **D** The YAP1 protein level upon NF1 knockdown by IHC staining. **E** Cells were left untreated or treated with MG-132, and western blots were performed to examine the indicated protein levels. **F** The YAP1 underwent stronger ubiquitination in the lv-shNF1 group, which was more apparent in the cells pretreated with MG132 (10 μM, 4 h)

To further investigate the relationship between NF1 and the localization of YAP1 in cancer promotion at the molecular level, YAP1 was examined in NOZ cells with NF1 knockdown. However, knockdown of NF1 in NOZ cells resulted in a significant reduction in the YAP1 protein level, but not in the mRNA level (Fig. 6A, B), suggesting that NF1 was involved in maintaining YAP1 protein stability. A cycloheximide (CHX)-chase assay was performed to evaluate the degradation of YAP1 in the NOZ cells with NF1 knockdown. After CHX treatment for four hours, endogenous YAP1degraded more rapidly in NF1 knockdown NOZ cells than in the control cells (Fig. 6C). IHC staining on tissue sections obtained from the CDX models revealed decreased YAP1 protein levels following NF1 knockdown (Fig. 6D). Treatment of NOZ cells with the proteasome inhibitor MG132 restored the protein level of YAP1 in NF1 knockdown NOZ cells (Fig. 6E). Furthermore, an increased number of high molecular weight smears, representing polyubiquitination of YAP1, were observed in the NF1 knockdown cells compared to the control cells, indicating the involvement of ubiquitination (Fig. 6F).

### Expression of an NF1 fragment could partially rescue YAP1 knockdown phenotypes

**Fig. 7:**
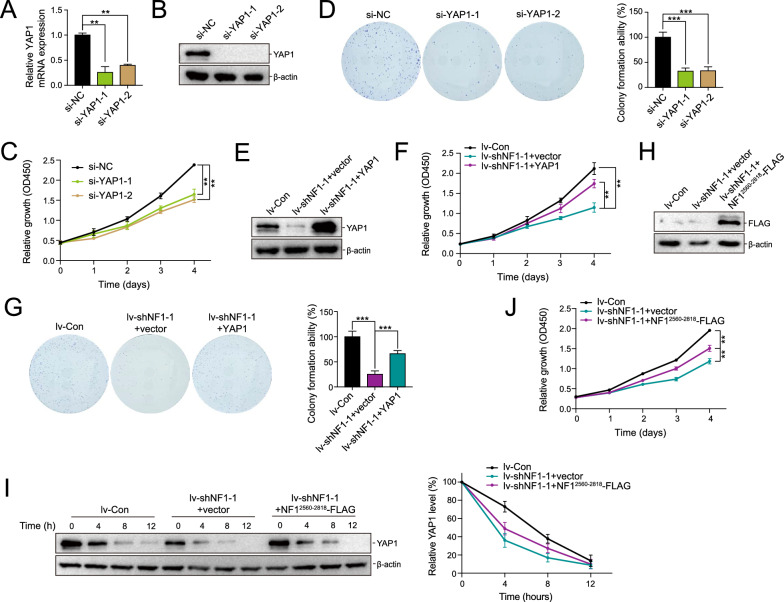
Overexpression of YAP1 and NF1^2560−2818^ could rescue growth ability partly in NOZ. **A** and **B** Knockdown of YAP1 in NOZ cell line was confirmed at the mRNA and protein levels. **C** and **D** CCK-8 and colony formation assays were applied to determine the proliferation of NOZ after YAP1 depletion.** E** The protein levels of YAP1 were detected in lv-Con, lv-shNF1-1 + vector, and lv-shNF1-1 + YAP1 groups.** F** and **G** CCK-8 and colony formation assays were applied to determine the proliferation of NOZ after YAP1 overexpression in lv-shNF1 cells.** H** Overexpression of NF1^2560−2818^ in the lv-shNF1 cell line was confirmed at the protein level by western blotting.** I** CHX chase assay was performed in the lv-Con, lv-shNF1 + vector, and lv-shNF1 + NF1^2560−2818^ group. **J** CCK-8 assays were applied to determine the proliferation of cells as in **H**

To investigate whether NF1 regulates the stability of YAP1 was critical to cell proliferation, siRNA was used to knock down YAP1 in NOZ cells, and the knockdown efficiency was verified through qRT-PCR and western blotting (Fig. 7A, B). The impact of YAP1 on NOZ cell proliferation was assessed using CCK-8 assay and colony formation (Fig. 7C, D). Cell proliferation was significantly inhibited upon YAP1 knockdown, in line with NF1 knockdown phenotypes.

Next, overexpression of YAP1 was applied to rescue the phenotype caused by NF1 knockdown (Fig. 7E). As expected, the proliferation and cell colonies were restored mainly by overexpression of YAP1 compared with empty vector-transfected lv-shNF1-1 NOZ cells (Fig. 7F, G). To further confirm the role of PPQY-WW interaction in YAP1 stabilization, the lv-shNF1-1 NOZ cells were transfected with NF1^2560−2818^-FLAG plasmid to overexpress the NF1 fragment that contains the PPQY motif. Western blot analysis confirmed the overexpression of the NF1 fragment (Fig. 7H). Moreover, the expression of the NF1^PPQY^ fragment partially rescued the proliferation of the lv-shNF1-1 knockdown NOZ cells (Fig. 7I). The CHX pulse-chase assay revealed that the NF1^2560−2818^-FLAG protein could rescue the proliferation, partly due to the stabilization of YAP1 (Fig. 7J).

### NF1 knockdown affected select YAP1 downstream genes in NOZ cells

**Fig. 8:**
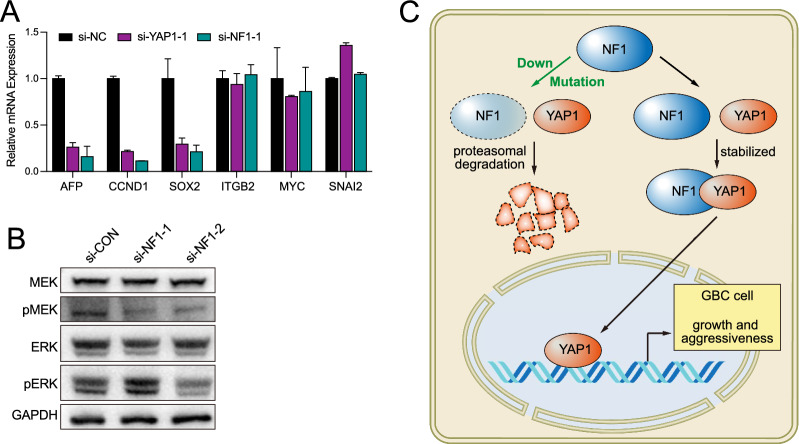
NF1 regulated the expression of YAP1 target genes. **A** The expression of YAP downstream target genes was analyzed by qPCR.** B** Western blot analysis of p-/MEK and p-/ERK. **C** Proposed model for the regulatory landscape of the NF1 and YAP1 in promoting the pathogenesis of GBC

To investigate whether the expression of YAP1 downstream genes can be regulated by NF1, six YAP1 downstream target genes (AFP, CCND1, SOX2, ITGB2, MYC, and SNAI2) were selected. These targets were identified based on the KEGG pathway database and our previous research, which demonstrated that YAP1 promotes gallbladder tumor growth by activating the AXL/MAPK pathway [34]. qPCR results showed that knockdown of NF1 led to downregulation of AFP, CCND1, and SOX2. In contrary, no significant changes were observed in ITGB2, MYC, and SNAI2 (Fig. 8A). Furthermore, investigation of the phosphorylation of AXL/MAPK pathway proteins showed no significant alterations due to NF1 knockdown, indicating that the observed effects on YAP1 downstream gene expression may be independent of Hippo pathway phosphorylation (Fig. 8B). These results suggested a potential role for NF1 in the regulation of specific YAP1 downstream targets independent of the RAS/MAPK signaling pathway. We proposed a model for the regulatory landscape of the NF1 and YAP1 in promoting the pathogenesis of GBC. Our model suggested that NF1 played an oncogenic role in GBC by interacting and stabilizing YAP1, which regulated the expression of the downstream target genes (Fig. 8C).

## Discussion

Our study discovered that NF1 unexpectedly acted as an oncogene in GBC by stabilizing YAP1, contradicting its previously observed protective effects. Despite commonly held beliefs that loss of NF1 promotes tumorigenesis through activation of the mitogen-activated protein kinase (MAPK) pathway [35], recent studies have shown that loss of NF1 accelerated liver tumor formation in mice, and low levels of NF1 messenger RNA were associated with shorter patient survival time [36]. Additionally, low NF1 expression was also associated with poor prognosis in colorectal cancer patients [24]. These findings highlight the importance of understanding the diverse functions of NF1 in cancer development and progression.

GBC is a prevalent and deadly type of gallbladder malignancy worldwide. YAP1, a transcriptional regulator, is widely activated in various human malignancies, including GBC [34, 37, 38]. Studies have shown that YAP/TAZ plays an essential role in cancer initiation or growth of most solid tumors [39, 40]. YAP1 interacts with the PPxY motif via its WW domains, leading to post-transcriptional modifications (PTMs) at different sites, contributing to its stability and spatial regulation [41]. For instance, the Large Tumor Suppressor Homolog 1 (LATS1) contains two PPxY motifs, and the latter PPPY^559^ motif in LATS1 is responsible for binding to both WW domains of YAP1, leading to the phosphorylation of YAP and regulation of its subcellular localization [42]. Similarly, the interaction between the first WW domain of YAP1 and the PPxY motif of ErbB4 enhances the transcription and translocation of the CTF of ErbB4, probably through phosphorylation of ErbB4 [43]. Additionally, the cytoplasmic retention and transcriptional suppression of YAP1 are mediated by its interaction with angiomotin (AMOT) and angiomotin-like 1 (AMOTL1) via the first WW domain of YAP1 [44–46]. Our study found that the NF1 PPxY motif (residues 2695–2698) could also interact with both YAP1 WW domains and affect its stability. Intriguingly, Neurofibromin 2 (NF2, also called Merlin) is an important member of Hippo signaling pathway [47, 48]. However, the amino acid sequence of NF2 does not contain a PPxY motif, and there is no evidence of interaction between YAP1 and NF2.

After conducting a comprehensive data mining analysis, we found that GBC samples exhibited higher levels of NF1 and YAP1 than normal tissues, which was associated with poor prognosis. in vitro and in vivo experiments showed that knocking down NF1 inhibited NOZ regarding proliferation and migration, resulting in a downregulation of YAP1 expression in vitro. Conversely, YAP1 knockdown impaired NOZ proliferation in vitro, whereas overexpression of YAP1 partially rescued the impaired proliferation ability in NF1 stably knockdown cells. Moreover, NF1 and YAP1 co-localized in vitro and interacted directly through the PPQY motif in NF1 and the WW domains of YAP1. Recent cryo-EM structures of NF1 showed that the PPQY motif was located within a loop at the C-terminal HEAT domains, allowing YAP1 to bind to it [16–18]. Our studies shed light on the molecular mechanism by which NF1 interacted with YAP1 and prevented its ubiquitination, thus increasing its stability.

However, several questions remain unsolved, including the phosphorylation status of YAP1 and the identification of the E3 ligase responsible for YAP1 ubiquitination. The absence of answers to these questions precludes the establishment of a direct causal relationship between NF1 and YAP1 ubiquitination. Additionally, technical difficulties have prevented the establishment of orthotopic xenograft models, and it is unclear why some other GBC cell lines express high levels of YAP1 and low levels of NF1 proteins. Moreover, we have unable to obtain lentiviruses that overexpress full-length NF1, which limits the study of NF1 carcinogenesis through gain-of-function. However, we did obtain a short NF1 fragment for rescue experiments to complement the NF1 knockdown phenotype. Notably, in NOZ and EH-GB1 cells, NF1 predominantly occurred in isoform 2, which carried a deletion mutation in the GRD domain, resulting in the loss of GTPase enzymatic activity. Our findings raised an intriguing question: Does NF1 truly lose its tumor suppressor role in all GBC cells? Although TCGA and GTEx database analysis revealed a significant correlation between NF1 and YAP1 in a variety of digestive tract-related malignancies, the role of NF1 in promoting or suppressing cancer in other solid tumors remains undetermined.

Herein, our study provided new insights into the function of NF1 in GBC and highlighted the importance of YAP1 stabilization in promoting GBC tumorigenesis. Further research is needed to fully elucidate the molecular mechanisms underlying NF1's oncogenic role in GBC and to explore potential therapeutic strategies targeting this pathway.

## Conclusions

In summary, our findings identified a novel regulatory mechanism for the oncogenic function of NF1 to regulate YAP stability through direct interaction between its PPQY motif and the WW domains of YAP1, even though NF1 was traditionally considered to be a tumor suppressor in RAS-MAPK signaling. A comprehensive analysis of our data revealed that NF1 protein could affect the stabilization and ubiquitination of YAP1, resulting in gallbladder cancer progression. Our study demonstrates that NF1 plays a different and crucial role in GBC progression, suggesting that NF1/YAP1 can be a potential therapeutic target for human GBCs.

## Supplementary Information

**Additional file 1: Table S1.** NF1/YAP1 expression scoring criteria in IHC.

**Additional file 2: Table S2**. Primer sequences for qPCR.

**Additional file 3: Figure S1.** Analysis of NF1 expression and mutation in digestive system tumors. **A** Expression level of NF1 gene in digestive system tumors and corresponding normal tissues. For the type of CHOL, LIHC, PAAD, and STAD in the TCGA project, the corresponding normal tissues of the GTEx database were included as controls. * *P *˂ 0.01. **B **NF1 mutations in CHOL, LIHC, PAAD, and STAD tumors by analysis of cBioPortal database.

**Additional file 4: Figure S2.** Cell proliferation and colony formation in EH-GB1 cells upon NF1 knockdown. **A** The mRNA expression level of NF1 in EH-GB1 cells transfected with lv-Con, lv -shNF1-1 and lv -shNF1-2. **B,** **C** CCK-8 assay (**B**) and colony formation assay (**C**) were applied to determine the proliferation of EH-GB1 after NF1 depletion.

**Additional file 5: Figure S3.** Model of the interactions between YAP1 tandem WW domains and NF1 PPQY motif as generated by AlphaFold. **A** Cartoon representation of NF1PPQY (yellow) in complex with YAP1WW1 (wheat). **B** Cartoon representation of NF1PPQY (sand) in complex with YAP1WW2 (teal).

**Additional file 6: Figure S4.** Correlation analysis between NF1 and YAP1 expression in CHOL, LIHC, PAAD, and STAD via GEPIA2.

**Additional file 7: Figure S5.** Representative immunofluorescence images of YAP1 (red) and NF1 (green) *in situ* revealed co-localization in NOZ cells (**A**) and EH-GB1 cells (**B**). Scale bar, 50 μm.

## Data Availability

All data generated or analyzed during this study are included in this published article and its additional files.

## Abbreviations

GBC: Gallbladder cancer
YAP1: Yes-associated protein 1
NF1: Neurofibromin 1
NF-1: Neurofibromatosis type-1 disease
qRT-PCR: Quantitative real-time PCR
WB: Western blot
IHC: Immunohistochemistry
Co-IP: Co-immunoprecipitation
ITC: Isothermal titration calorimetry assay
CHX: Cycloheximide
GTP: Guanosine triphosphate
GDP: Guanosine diphosphate
OS: Overall survival
GRD: GTPase-activating protein-related domain
MPNST: Malignant peripheral nerve sheath tumors
GIST: Gastrointestinal stromal tumor
CHOL: Cholangiocarcinoma
LIHC: Liver hepatocellular carcinoma
PAAD: Pancreatic adenocarcinoma
STAD: Stomach adenocarcinoma
COSMIC database: Catalogue of Somatic Mutations in Cancer
cryo-EM: Cryo-electron microscopy
MAPK: Mitogen-activated protein kinase
PTMs: Post-transcriptional modifications
LATS1: Large tumor suppressor homolog 1
AMOT: Angiomotin
AMOTL1: Angiomotin-like 1
NF2: Neurofibromin 2
PFA: Paraformaldehyde
AOD: Average optical density
