# Supplementary material for: The oncogenic role of NF1 in gallbladder cancer through regulation of YAP1 stability by direct interaction with YAP1
Source: J Transl Med. 2023 May 5;21:306. doi: 10.1186/s12967-023-04157-9 (PMC10163693; doi:10.1186/s12967-023-04157-9)
Supplement: Supplementary file 1 — Additional file 1: Table S1. NF1/YAP1 expression scoring criteria in IHC. [file 12967_2023_4157_MOESM1_ESM.pdf]

Additional file

The oncogenic role of NF1 in gallbladder cancer through regulation of YAP1 stability by direct interaction with YAP1

Lingxiao Zhang, Lin Jiang, Ling Zeng, Zhaohui Jin, Xuanjia Dong, Yuhan Zhang, Litian Chen, Yijun Shu, Yingbin Liu, Ying Huang

Table S1. NF1/YAP1 expression scoring criteria in IHC

Intensity of protein expression	Proportion	Score	Figure
Weakly positive	0	0	1C, 5E
Moderately positive	1-25%	1	
Strongly positive	26-50%	2	
No stronger intensity	51-75%	3	
Negative	76-100%	4	
