# Supplementary material for: The oncogenic role of NF1 in gallbladder cancer through regulation of YAP1 stability by direct interaction with YAP1
Source: J Transl Med. 2023 May 5;21:306. doi: 10.1186/s12967-023-04157-9 (PMC10163693; doi:10.1186/s12967-023-04157-9)
Supplement: Supplementary file 2 — Additional file 2: Table S2. Primer sequences for qPCR. [file 12967_2023_4157_MOESM2_ESM.pdf]

**Table S2**. Primer sequences for qPCR

| Primer | Forward (5' - 3') | Reverse (3' - 5') | Figure |
|---|---|---|---|
| NF1-q1 | GCGGCCTCACTACTATTTTAAAG | ATCTAATCTCATTGTGTCCTTTGG | 4A, 4B |
| NF1-q2 | CTGGAAAAATGTCTTGCTGGG | AGAGGCAGAATTCCGAAGTTC | 4A, 4B |
| NF1-q3 | GTTTCACTTCTAGCTGGTCTCC | CGCACTTTCATCTTCAACTTCAC | 4A, 4B |
| NF1-q4 | CCACTCCCTACTGAATAAAGCTAC | ATACGGTGAGACAATGGCAG | 4A, 4B |
| NF1-q5 | TGTGCCACTGTTTATACCAGG | CACTTCCTACTGCACCGATG | 4A, 4B |
| NF1-q6 | CGGAAGGGAAAAGGGAACTC | AGAACAGCTTCAGTGTCAGG | 2A, 2C, 3A |
| NF1-q7 | GTTTCACTTCTAGCTGGTCTCC | CGCACTTTCACTTCAACTTCAC | 2A, 2C, 3A |
| NF1-q8 | AGTGTCTCATGGGCAGATAAAG | GAAGTGGCTGTAATTTGGTTAGTG | 2A, 2C, 3A |
| YAP1-q1 | ACAAGCATGACTCAGGATG | TGTTTCACTGGAGCACTCTG | 6A, 7A |
| YAP1-q2 | AGATGGAGAAGGAGAGGCTG | AGTGTTGGTAACTGGCTACG | 6A, 7A |
| YAP1-q3 | CCCAGATGACTTCCTGAACAG | CCATCTCCTTCCAGTGTTCC | 6A, 7A |
| AFP | TCAGCAGCTTGGTGGTGGATG | CCCTGAGCTTGGCACAGATCC | 8A |
| CCND1 | CATGCTGAAGGCGGAGGAGAC | CCAGGTGGCGACGATCTTCC | 8A |
| SOX2 | GCCCAGGAGAACCCCAAGATG | GCAGCCGCTTAGCCTCGTC | 8A |
| ITGB2 | GTGGATGAGAGCCGAGAGTGTG | GAGCCTTCCAGATGACCAGCAG | 8A |
| MYC | CCGCCCCTGTCCCCTAGC | CCACCGCCGTCGTTGTCTC | 8A |
| SNAI2 | GACTACCGCTGCTCCATTCCAC | GGACTCACTCGCCCCAAAGATG | 8A |
| GAPDH | GGAGCGAGATCCCTCCAAAAT | GGCTGTTGTCATACTTCTCATGG | ALL |
