# Supplementary material for: The oncogenic role of NF1 in gallbladder cancer through regulation of YAP1 stability by direct interaction with YAP1
Source: J Transl Med. 2023 May 5;21:306. doi: 10.1186/s12967-023-04157-9 (PMC10163693; doi:10.1186/s12967-023-04157-9)
Supplement: Supplementary file 3 — Additional file 3: Figure S1. Analysis of NF1 expression and mutation in digestive system tumors. A Expression level of NF1 gene in digestive system tumors and corresponding normal tissues. For the type of CHOL, LIHC, PAAD, and STAD in the TCGA project, the corresponding normal tissues of the GTEx database were included as controls. * P ˂ 0.01. B NF1 mutations in CHOL, LIHC, PAAD, and STAD tumors by analysis of cBioPortal database. [file 12967_2023_4157_MOESM3_ESM.pdf]

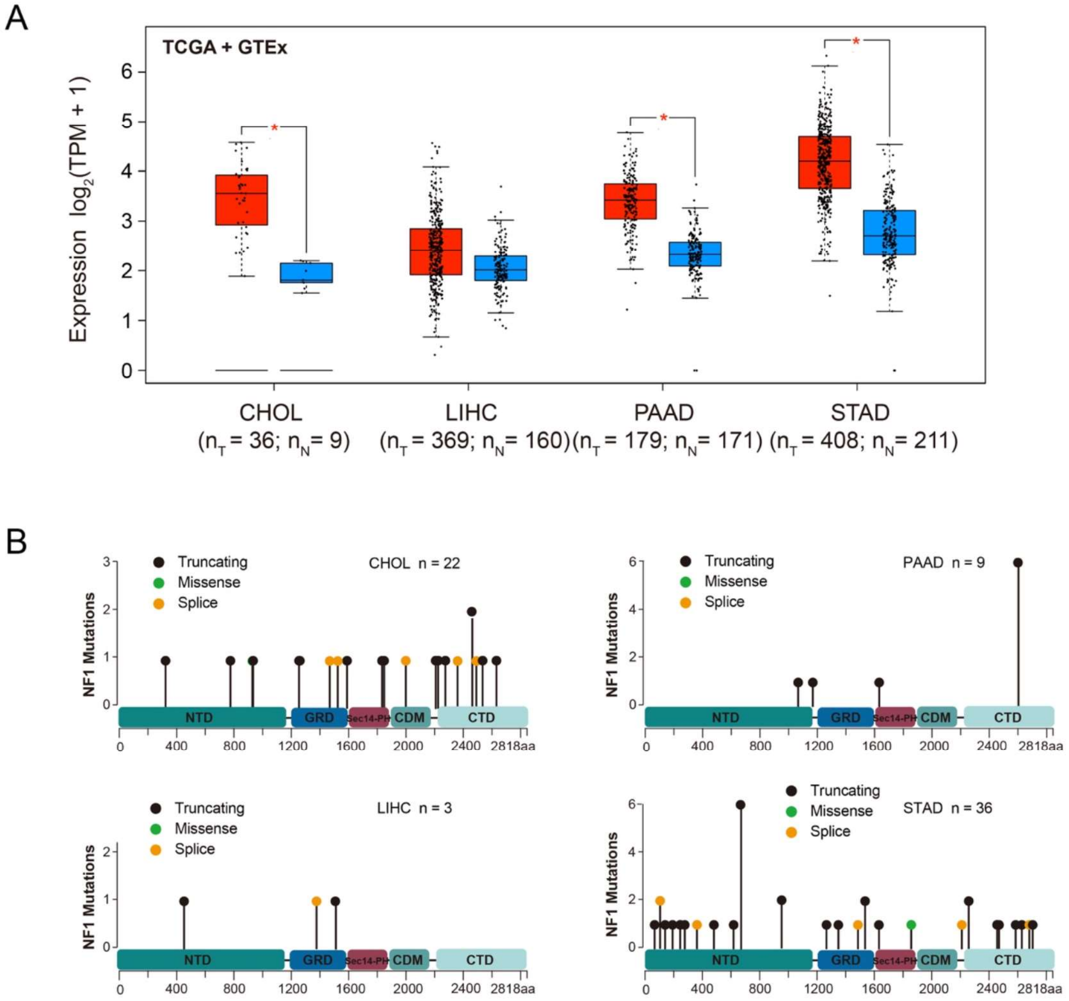

Figure S1. Analysis of NF1 expression and mutation in digestive system tumors. **A** Expression level of NF1 gene in digestive system tumors and corresponding normal tissues. For the type of CHOL, LIHC, PAAD, and STAD in the TCGA project, the corresponding normal tissues of the GTEx database were included as controls. * $P < 0.01$. **B** NF1 mutations in CHOL, LIHC, PAAD, and STAD tumors by analysis of cBioPortal database.
