# Supplementary material for: The oncogenic role of NF1 in gallbladder cancer through regulation of YAP1 stability by direct interaction with YAP1
Source: J Transl Med. 2023 May 5;21:306. doi: 10.1186/s12967-023-04157-9 (PMC10163693; doi:10.1186/s12967-023-04157-9)
Supplement: Supplementary file 4 — Additional file 4: Figure S2. Cell proliferation and colony formation in EH-GB1 cells upon NF1 knockdown. A The mRNA expression level of NF1 in EH-GB1 cells transfected with lv-Con, lv -shNF1-1 and lv -shNF1-2. B, C CCK-8 assay (B) and colony formation assay (C) were applied to determine the proliferation of EH-GB1 after NF1 depletion. [file 12967_2023_4157_MOESM4_ESM.pdf]

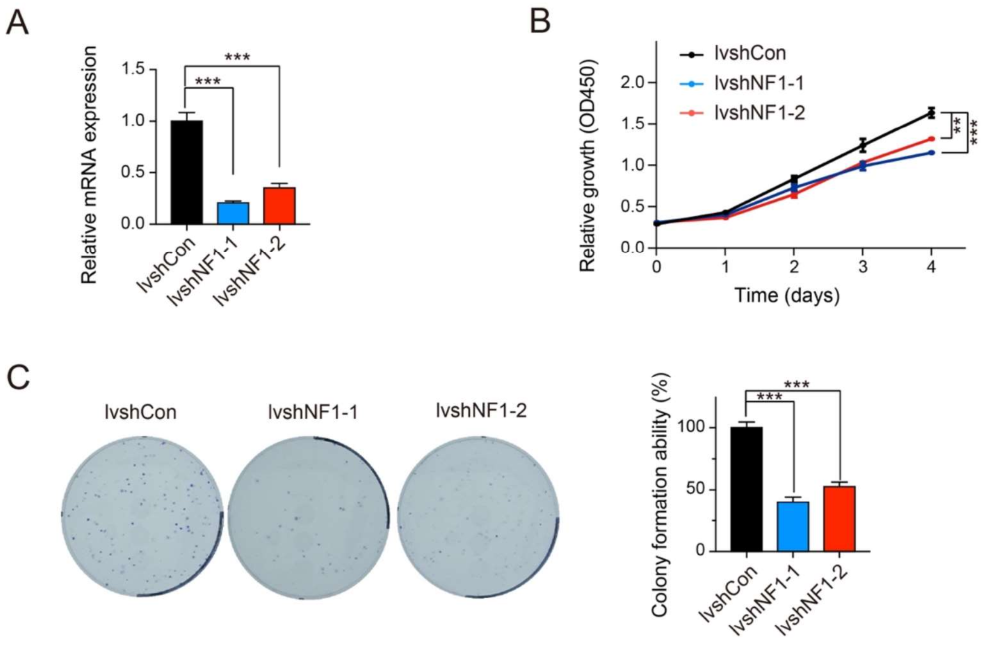


**Figure S2.** Cell proliferation and colony formation in EH-GB1 cells upon NF1 knockdown. **A** The mRNA expression level of NF1 in EH-GB1 cells transfected with lv-Con, lv -shNF1-1 and lv -shNF1-2. **B, C** CCK-8 assay (**B**) and colony formation assay (**C**) were applied to determine the proliferation of EH-GB1 after NF1 depletion.
