# Supplementary material for: The oncogenic role of NF1 in gallbladder cancer through regulation of YAP1 stability by direct interaction with YAP1
Source: J Transl Med. 2023 May 5;21:306. doi: 10.1186/s12967-023-04157-9 (PMC10163693; doi:10.1186/s12967-023-04157-9)
Supplement: Supplementary file 5 — Additional file 5: Figure S3. Model of the interactions between YAP1 tandem WW domains and NF1 PPQY motif as generated by AlphaFold. A Cartoon representation of NF1PPQY (yellow) in complex with YAP1WW1 (wheat). B Cartoon representation of NF1PPQY (sand) in complex with YAP1WW2 (teal). [file 12967_2023_4157_MOESM5_ESM.pdf]

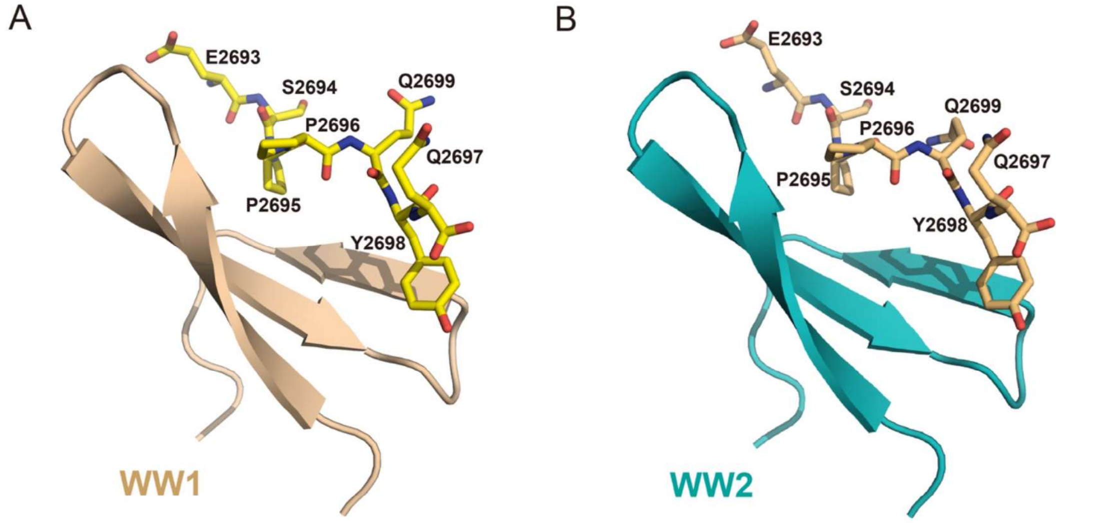

Figure S3. Model of the interactions between YAP1 tandem WW domains and NF1 PPQY motif as generated by AlphaFold. **A** Cartoon representation of $NF1^{PPQY}$ (yellow) in complex with $YAP1^{WW1}$ (wheat). **B** Cartoon representation of $NF1^{PPQY}$ (sand) in complex with $YAP1^{WW2}$ (teal).
