# Supplementary material for: The oncogenic role of NF1 in gallbladder cancer through regulation of YAP1 stability by direct interaction with YAP1
Source: J Transl Med. 2023 May 5;21:306. doi: 10.1186/s12967-023-04157-9 (PMC10163693; doi:10.1186/s12967-023-04157-9)
Supplement: Supplementary file 6 — Additional file 6: Figure S4. Correlation analysis between NF1 and YAP1 expression in CHOL, LIHC, PAAD, and STAD via GEPIA2. [file 12967_2023_4157_MOESM6_ESM.pdf]

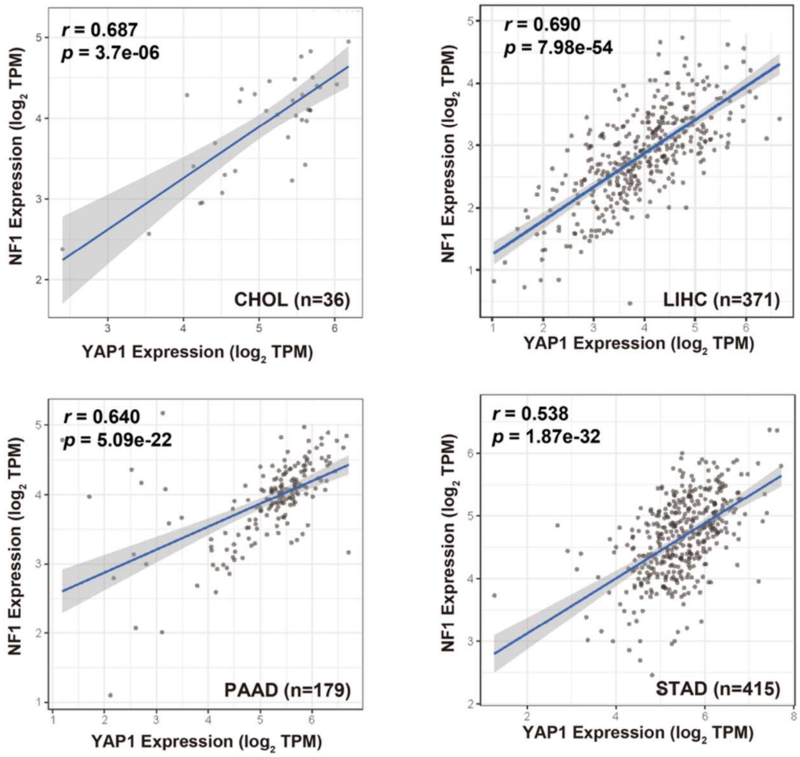


**Figure S4.** Correlation analysis between NF1 and YAP1 expression in CHOL, LIHC, PAAD, and STAD via GEPIA2.
