# Supplementary material for: The oncogenic role of NF1 in gallbladder cancer through regulation of YAP1 stability by direct interaction with YAP1
Source: J Transl Med. 2023 May 5;21:306. doi: 10.1186/s12967-023-04157-9 (PMC10163693; doi:10.1186/s12967-023-04157-9)
Supplement: Supplementary file 7 — Additional file 7: Figure S5. Representative immunofluorescence images of YAP1 (red) and NF1 (green) in situ revealed co-localization in NOZ cells (A) and EH-GB1 cells (B). Scale bar, 50 μm. [file 12967_2023_4157_MOESM7_ESM.pdf]

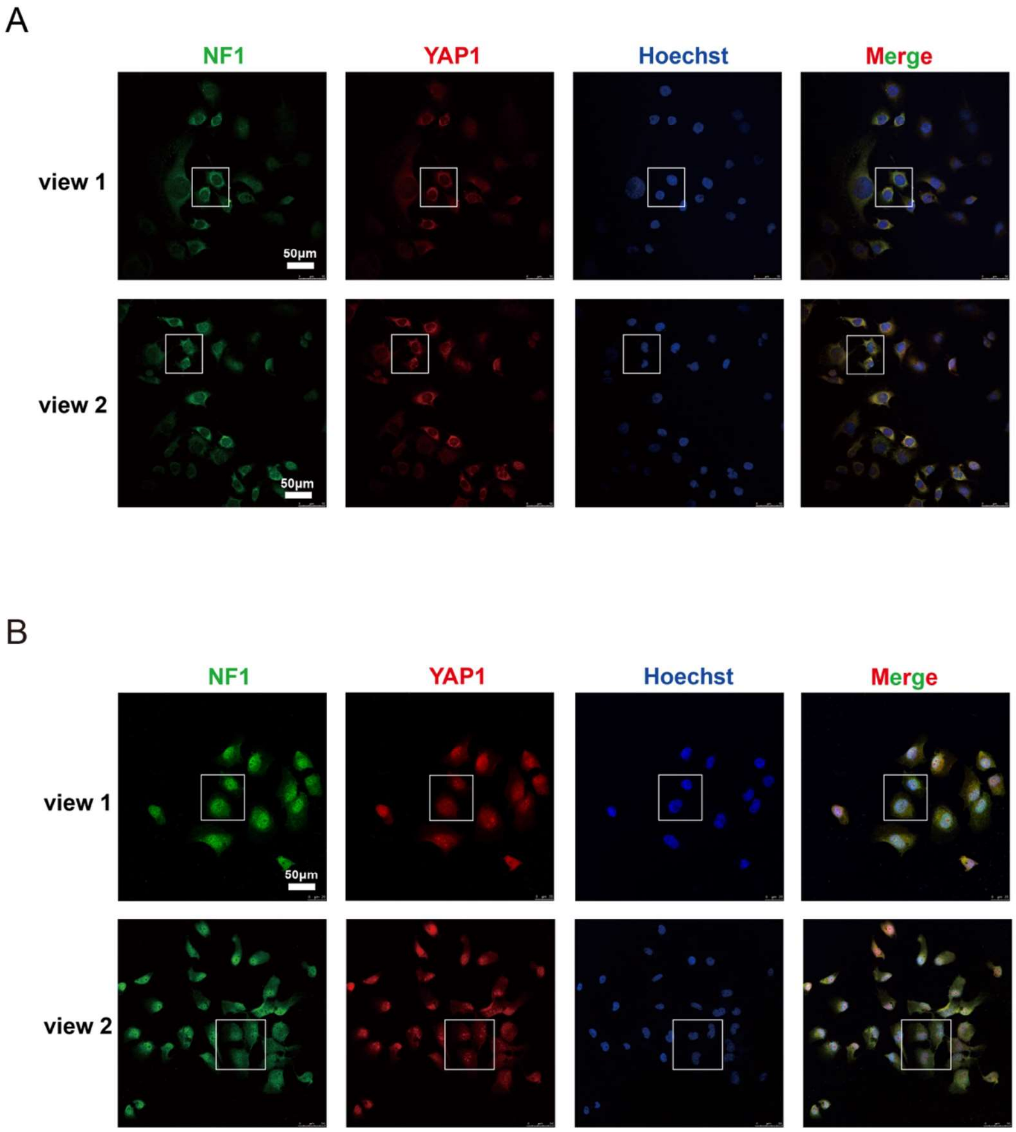

Figure S5. Representative immunofluorescence images of YAP1 (red) and NF1 (green) *in situ* revealed co-localization in NOZ cells (**A**) and EH-GB1 cells (**B**). Scale bar, 50 μm.
